# Region- and time- specific effects of ketamine on cerebral blood flow: a randomized controlled trial

**DOI:** 10.1038/s41386-023-01605-4

**Published:** 2023-05-25

**Authors:** Matti Gärtner, Anne Weigand, Marvin Sören Meiering, David Weigner, Luisa Carstens, Christian Keicher, Rita Hertrampf, Christian Beckmann, Maarten Mennes, Andreas Wunder, Simone Grimm

**Affiliations:** 1 Medical School Berlin, Berlin, Germany; 2 Department of Psychiatry and Psychotherapy, Charité, Universitätsmedizin Berlin, Corporate Member of Freie Universität Berlin and Humboldt-Universität Zu Berlin, Berlin, Germany; 3 Charité Research Organisation GmbH, Berlin, Germany; 4 SBGneuro Ltd., Oxford, UK; 5 Translational Medicine and Clinical Pharmacology, Boehringer Ingelheim Pharma GmbH & Co. KG, Biberach an der Riss, Germany; 6 Department of Psychiatry, Psychotherapy and Psychosomatics, University Hospital of Psychiatry, University of Zurich, Zurich, Switzerland

**Keywords:** Neuroscience, Drug discovery

## Abstract

There is intriguing evidence suggesting that ketamine might have distinct acute and delayed neurofunctional effects, as its acute administration transiently induces schizophrenia-like symptoms, while antidepressant effects slowly emerge and are most pronounced 24 h after administration. Studies attempting to characterize ketamine’s mechanism of action by using blood oxygen level dependent (BOLD) imaging have yielded inconsistent results regarding implicated brain regions and direction of effects. This may be due to intrinsic properties of the BOLD contrast, while cerebral blood flow (CBF), as measured with arterial spin labeling, is a single physiological marker more directly related to neural activity. As effects of acute ketamine challenge are sensitive to modulation by pretreatment with lamotrigine, which inhibits glutamate release, a combination of these approaches should be particularly suited to offer novel insights. In total, 75 healthy participants were investigated in a double blind, placebo-controlled, randomized, parallel-group study and underwent two scanning sessions (acute/post 24 h.). Acute ketamine administration was associated with higher perfusion in interior frontal gyrus (IFG) and dorsolateral prefrontal cortex (DLPFC), but no other investigated brain region. Inhibition of glutamate release by pretreatment with lamotrigine abolished ketamine’s effect on perfusion. At the delayed time point, pretreatment with lamotrigine was associated with lower perfusion in IFG. These findings underscore the idea that regionally selective patterns of CBF changes reflect proximate effects of modulated glutamate release on neuronal activity. Furthermore, region- specific sustained effects indicate both a swift restoration of disturbed homeostasis in DLPFC as well changes occurring beyond the immediate effects on glutamate signaling in IFG.

## Introduction

The NMDA receptor antagonist ketamine not only transiently induces schizophrenia-like positive and cognitive symptoms, but also rapidly reduces depressive symptoms in otherwise treatment-resistant patients [1]. Interestingly, positive symptoms of schizophrenia appear before antidepressant effects emerge, thereby indicating distinct acute and delayed neurofunctional effects of ketamine. Imaging studies attempting to characterize ketamine’s mechanism of action by using blood oxygen level dependent (BOLD) imaging during resting state conditions or during emotional and cognitive tasks have yielded inconsistent results regarding implicated brain regions and direction of effects [2]. Discrepancies regarding ketamine’s effects on the brain may at least in part be due to intrinsic properties of the BOLD contrast, which measures a complex signal indirectly related to neural activity [3, 4]. In contrast, regional cerebral blood flow, as measured with positron emission tomography (PET) or arterial spin labeling (ASL), is a single physiological marker reflecting metabolic activity, which is more directly related to neuronal activity within a given region [5]. ASL-derived perfusion has mainly been used to investigate cerebrovascular diseases, dementia, and neuro-oncological disorders, but it is gaining traction in psychiatry and other fields of neuroscience as a research tool [6]. ASL uses a magnetic pulse to label blood as it perfuses through the brain to provide oxygen and nutrients to tissue [7]. As it exhibits a temporally stable and relatively straightforward signal to interpret and has been demonstrated as sensitive for detecting drug effects [8–10], ASL may offer novel insights into ketamine’s mechanism of action.

During acute administration, ketamine was shown to have an immediate effect on CBF with increases in prefrontal and cingulate cortices, as well as in subcortical regions such as thalamus [11–15], but a decrease in the hippocampus [8, 14, 16]. Perfusion increases measured with ASL are consistent with the reported increases in fluorodeoxyglucose uptake in similar areas, which suggests that changes in CBF reflect proximate effects of ketamine-induced changes in neuronal activity and thus in glucose metabolism [17–19]. Few studies investigated the prolonged impact of ketamine administration and reported that CBF changes diminished after 4 h in healthy participants [8, 11]. In depressive patients, findings indicate delayed effects of ketamine by demonstrating an increase in thalamus and cingulate perfusion 24 h after ketamine administration, while serial infusion therapy was associated with CBF decreases in hippocampus and right insula [20–22].

Complementary insights into ketamine’s mechanism of action might be provided by direct modulatory approaches. Accordingly, several studies have demonstrated that effects of acute ketamine challenge are sensitive to modulation by pretreatment with lamotrigine, a broad-spectrum anticonvulsant that inhibits voltage-gated ion channels, with downstream effects resulting in inhibition of glutamate release [23]. In a recent systematic review, Veraart et al. [24] reported that until now seven studies were conducted on the effects of lamotrigine prior to ketamine administration and that one of these also measured CBF. Some of the results are conflicting, with reports of a significant attenuation of ketamine-induced psychotomimetic effects and cognitive impairments by pretreatment with lamotrigine [25, 26] as well as of no effect [27] on these measures. However, regarding resting-state blood oxygenation level-dependent (BOLD) responses and global brain connectivity during acute ketamine administration, studies consistently show an attenuation by lamotrigine pretreatment [26–28]. Since lamotrigine pretreatment had no effect on resting brain perfusion [15], it was proposed that the attenuation of ketamine’s acute effects by lamotrigine might not be due to changes in neurovascular responsivity but rather to reduced glutamate release.

However, previous studies investigating the impact of lamotrigine pretreatment were conducted during the acute administration of ketamine and it is not yet known whether the inhibition of glutamate release via lamotrigine has longer term consequences. Along that line, previous studies investigating the impact of ketamine on perfusion were conducted either during or after its administration, but no study has yet investigated both acute and delayed effects in the same participants. Longitudinal assessments of participants would however provide additional insights, given that psychotomimetic effects appear during the administration and quickly diminish, while antidepressant effects of ketamine are most pronounced 24 h after administration, thereby indicating sustained adaptive changes in brain dynamics [29].

Consequently, the aim of the present study was to investigate acute and delayed (24 h) effects of a single dose of ketamine on resting brain perfusion in predefined brain regions. To determine whether these are modulated by inhibited glutamate release, we also investigated the impact of lamotrigine prior to ketamine administration.

## Methods

### Participants

Healthy, right-handed male and female participants (18–45 years) were recruited in a double blind, placebo-controlled, randomized, single dose, parallel-group study with three treatment conditions (placebo-placebo, placebo-ketamine, lamotrigine-ketamine). Exclusion criteria were a history of or current psychiatric conditions, as determined by the SCID-5-CV at screening, a positive drug screen, alcohol or substance dependence within the last 12 months, prescribed psychotropic medication within 28 days prior to screening and non-prescription medication within 48 h prior to treatment visit. Further exclusion criteria were a history of relevant neurological diseases, migraine headaches, relevant medical condition, MRI exclusion criteria, and pregnancy. All participants gave written consent to participate in the study, which was approved by the local ethics committee and registered at ClinicalTrials.gov (NCT04156035).

### Experimental design & procedure

All eligible participants were randomly assigned to one of three treatment groups in a 1:1:1 ratio. Participants in the first group were pretreated with placebo and received a placebo infusion (placebo-placebo group, PP). Participants in the second group were pretreated with placebo and received a ketamine infusion (placebo-ketamine group, PK), and participants in the third group were pretreated with lamotrigine and received ketamine (lamotrigine-ketamine group, LK). All participants underwent two scanning sessions on two consecutive days. Before the first scanning session, participants were pretreated with an oral dose of 300 mg lamotrigine (LK) or matching placebo (PP, PK) 2 hours before the scanning procedures. During the first scanning session (acute), participants were intravenously administered ketamine or placebo (ketamine dosage: 0.12 ± 0.003 mg/kg during the first minute followed by a continuous infusion of approximately 0.31 mg/kg/h). Blood samples were taken 55 minutes after commencing ketamine infusion to determine ketamine plasma levels. Before the infusion started, all participants underwent a short resting state fMRI scan, that was repeated after the start of the infusion. Next, participants performed a picture viewing and an emotional working memory task (reported elsewhere; [30]). The scanning session ended with the ASL sequence reported here. Total scanning time was approximately 1 h. To investigate the possible delayed effects of a single dose of ketamine on perfusion, participants underwent the same scanning procedure without the drug treatment and without the baseline resting state scan 24 h later. A more detailed description of the experimental design and procedures is provided in a previous publication [30], and in the Supplementary Methods.

### Materials

#### Psychometric assessments

Psychometric assessments were conducted after both scanning sessions. Dissociative symptoms were assessed using the Dissociation-Tension-Scale (DSS; Stiglmayr et al. [31]), which assess dissociative phenomena on a psychological, somatoform and global scale. Altered states of consciousness were assessed using the 5D Altered States of Consciousness Scale (5D-ASC; Dittrich, [32]). The 5D-ASC assesses altered states of consciousness on 5 main dimensions: oceanic boundlessness (OBN), dread of ego dissolution (DED), visionary restructuralization (VRS), auditory alterations (AUA), and vigilance reduction (VIR). Participants use a visual analog scale to report the extent to which the experiences during the infusion differ from their normal waking state. Further, the mood state was assessed prior and after each scanning session using the German version of the Positive and Negative Affect Schedule (PANAS; Breyer & Bluemke, [33]; Krohne et al. [34]; Watson et al. [35]). The questionnaire consists of 20 adjectives that describe different sensations and feelings. 10 adjectives each capture the dimensions *positive affect* (PA) and *negative affect* (NA).

### Data acquisition and analysis

Acquisition of brain images was conducted using a 3 Tesla MRI scanner (PRISMA, Siemens Medical Systems, Erlangen, Germany) with a 64-channel head coil at the Berlin Center for Advanced Neuroimaging (BCAN). An anatomical brain image was acquired with a 3D T1-weighted scan (Magnetization Prepared Rapid Acquisition Gradient Echo sequence, TE = 3.03 ms, TR = 2.3 s, 192 slices and FOV = 256 × 256 × 192 mm). ASL data was acquired using a multi-post labeling delay pseudo continuous [36] ASL sequence (labeling duration = 1800 ms, post labeling delays = 400,700,1000,1300,1600,1900,2200 and 2500 ms, labeling plane positioned 90 mm below the center of the imaging region, background suppression using pre-saturation and two inversion pulses timed as per Günther et al. [37] with T1_opt_ = 700 ms, with nulling occurring 100 ms prior to excitation) with a segmented 3D-GRASE readout (4 segments, Resolution=3.4 × 3.4 × 3.3 mm, 38 slices, TR = 5 s, TE = 35 ms, 120° refocusing flip angle, left-right phase-encoding, 6/8 slice partial Fourier, 1 label/control pair per post labeling delay). Two separate calibration (M0) scans were also acquired with no ASL labeling or background suppression pulses using identical readout parameters: one with left-right and one with right-left phase-encoding to allow for B0-induced distortion correction. Total ASL scan time was 6 min 8 s. The ASL data were preprocessed using FSL tools, correcting for motion [38], B0-distortion [39] and coil sensitivity non-uniformity (by comparing the calibration images with and without pre-scan normalize corrections) before taking the control-label difference at each post labeling delay. Kinetic model fitting [40], accounting for a macrovascular component [41], was performed using a spatial Bayesian prior to stabilize the fitting process [42, 43]. Finally, relative perfusion (rCBF) was calculated relative to global CBF, with the added constraint that global CBF was restricted to a gray matter mask. Analysis of rCBF allows to further isolate regional changes in CBF from global CBF changes.

**Fig. 1:**
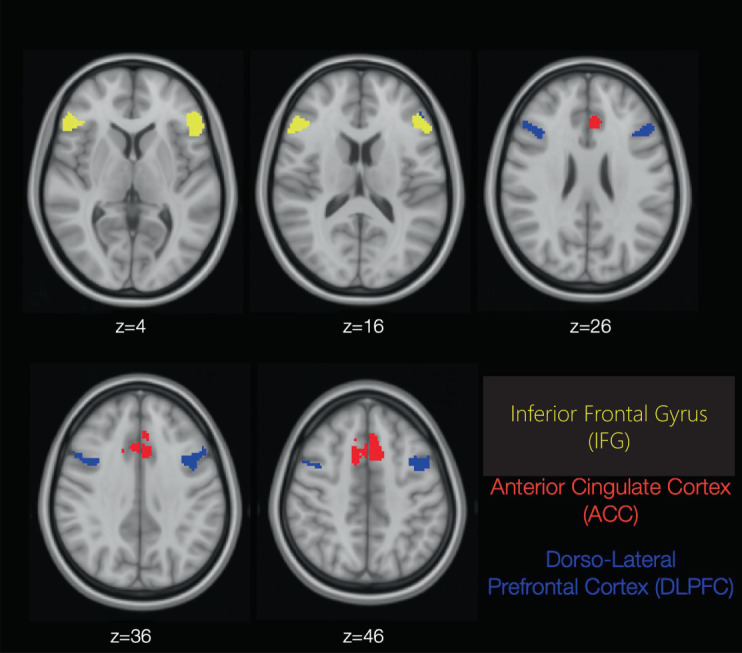
Regions of interest (ROIs). The three bilateral ROIs used in the main data analysis are shown on axial brain slices in the respective plane.

From the preprocessed ASL images, mean CBF scores were extracted from the following three prespecified bilateral regions of interest (ROIs): inferior frontal gyrus (IFG), dorsolateral prefrontal cortex (DLPFC), and anterior cingulate cortex (ACC). See Fig. 1 and Supplementary Table 2 for a detailed ROI description. The ROIs were derived from activation maps obtained in an independent sample of 15 healthy controls. Activation maps were intersected with substructures of the Harvard-Oxford atlas implemented in FSL to obtain anatomical specificity (see Supplementary Methods for details). This ROI definition ensured focus on those subregions within the larger atlas structures that exhibit the strongest task-induced activations during the fMRI tasks that were part of the study and are reported elsewhere [30]. The reason for using these ROIs also for the ASL data was to ensure comparability of results between the different MRI modalities collected in our studies.

Exploratively, three additional bilateral ROIs were investigated: amygdala, hippocampus, and thalamus. These ROIs were defined separately for each participant using the outcome of an automated subcortical segmentation via FSL FIRST.

### Statistical analyses

Univariate ANOVAs with the main factor group (PK, LK, PP) were performed for the psychometric measures and the acute rCBF data. The data from the left and right hemispheres was averaged for each ROI. To correct for multiple testing, a Bonferroni adjusted alpha level of α = 0.0167 (α = 0.05/3) was applied to the analysis of the three ROIs of main interest. In case of a significant main effect of group, Tukey’s post hoc tests were performed, and for the rCBF data the following additional analyses were conducted: 1. To investigate prolonged effects, a univariate ANOVA at the delayed time point was conducted. 2. To investigate effects of laterality, univariate ANOVAs for left and right ROIs were conducted separately. 3. To investigate differences between the two time points within groups, paired *t* tests were conducted. Correlation analyses were conducted using Pearson’s correlation coefficient. All statistical analyses were conducted using SPSS version 27 (IBM, USA).

## Results

75 male and female participants (age: M = 28.96, SD = 6.58) complete the study and were randomly assigned to one of the three treatment conditions in a 1:1:1 ratio: placebo-placebo (PP, *n* = 25), placebo-ketamine (PK, *n* = 25), placebo-lamotrigine (LK, *n* = 25). The final sample for the analysis of the CBF data consisted of 68 male and female participants (age: *M* = 28.57, SD = 6.27). Seven participants had to be excluded due to insufficient data quality. One participant was excluded due to motion. The others were excluded due to large changes in slice prescriptions between the different ASL sub-scans, this renders the data unanalyzable. The final group sizes for the three treatment groups were as follows: placebo-ketamine (PK, *n* = 23), placebo-lamotrigine (LK, *n* = 23), and placebo-placebo (PP, *n* = 22).

No difference in ketamine plasma concentration was found between the PK and LK group (*T*(44) = 1.62, *p* = 0.11). Univariate ANOVAs calculated for PANAS change scores (post-pre) showed no significant between-group differences for Δ-positive affect score (*F*(2, 65) = 1.6, *p* = 0.21) and Δ-negative affect score (*F*(2, 65) = 0.18, *p* = 0.83) at day 1. On a descriptive level Δ-positive affect scores increased slightly after the infusion in the PK group (*ΔM* 0.65 *SD* 2.5) and LK group (*ΔM* 0.57 *SD* 3.45) and decreased in the PP group (*ΔM* −0.64 *SD* 1.84). On the DSS and the ASC scales the PP group had lower scores compared to the PK and LK groups (all *p* < 0.001), and no differences were observed between the PK and LK groups. No between-group differences were observed at day 2 (all *p* > 0.9). A more detailed description of the demographic data and the psychometric results is provided in a previous publication [30] and in Supplementary Table 3.

The univariate ANOVAs conducted for rCBF data at the acute time point showed a significant effect of group for the bilateral IFG (*F*(2, 65) = 6.59, *p* = 0.002, η² = 0.17), and bilateral DLPFC (*F*(2, 65) = 4.73, *p* = 0.012, η² = 0.13). No significant effect of group was observed for the bilateral ACC (*F*(2, 65) = 0.75, *p* = 0.48). Furthermore, the three additionally investigated bilateral ROIs (amygdala, hippocampus, and thalamus) showed no significant effect of group (all *p* > 0.05).

Post hoc tests conducted for the bilateral IFG showed that rCBF was stronger in the PK group compared to LK (*M*_*PK*_ = 113.17, *SD*_*PK*_ = 11.47, *M*_*LK*_ = 103.58, *SD*_*LK*_ = 8.50, *p* = 0.003, 95% CI [2.94, 16.24]) and compared to PP (*M*_*PP*_ = 105.68, *SD*_*PP*_ = 7.76, *p* = 0.025, 95% CI [0.77, 14.22]). Post hoc tests conducted for the bilateral DLPFC showed that rCBF was stronger in the PK group compared to LK (*M*_*PK*_ = 103.61, *SD*_*PK*_ = 6.79, *M*_*LK*_ = 97.11, *SD*_*LK*_ = 7.96, *p* = 0.016, 95% CI [1.03, 11.96]) and compared to PP (*M*_*PP*_ = 98.05, *SD*_*PP*_ = 8.38, *p* = 0.048, 95% CI [0.03, 11.07]).

**Fig. 2:**
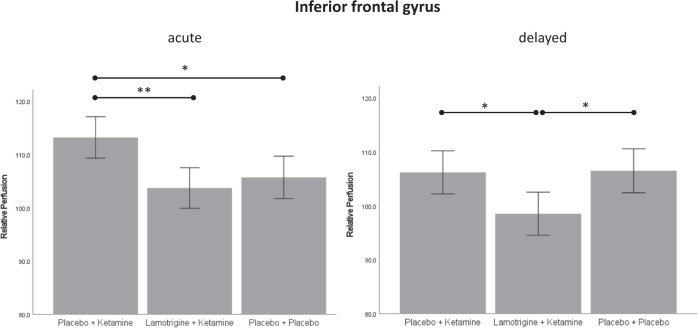
Relative perfusion in the inferior frontal gyrus. Perfusion shown for the acute time point (left) and the delayed time point (right). * = significant at *p* < 0.05; ** = significant at *p* < 0.01.

**Fig. 3:**
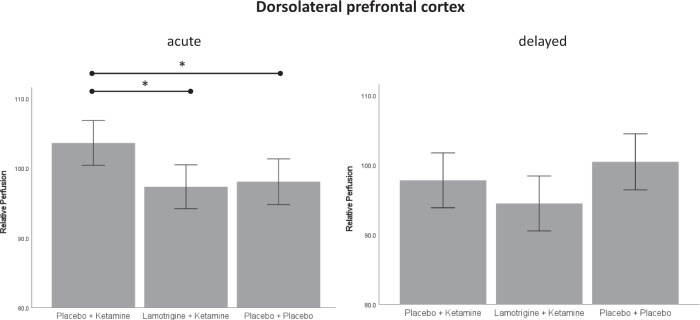
Relative perfusion in the dorsolateral prefrontal cortex. Perfusion shown for the acute time point (left) and the delayed time point (right). * = significant at *p* < 0.05.

At the delayed time point, a significant effect of group was observed for the bilateral IFG (*F*(2, 65) = 6.01, *p* = 0.004, η² = 0.16), but not for the bilateral DLPFC (*F*(2, 65) = 2.40, *p* = 0.099). Post hoc comparisons conducted for the bilateral IFG showed that rCBF was lower in the LK group compared to PK (*M*_*LK*_ = 99.33, *SD*_*LK*_ = 8.30, *M*_*PK*_ = 107.03, *SD*_*PK*_ = 9.84, *p* = 0.013, 95% CI [1.38, 14.03]) and compared to PP (*M*_*PP*_ = 107.49, *SD*_*PP*_ = 8.59, *p* = 0.009, 95% CI [1.77, 14.56]). Acute and delayed effects for the IFG and DLPFC are shown in Figs. 2 and 3.

At the acute time point, a significant effect of group was observed for the right IFG (*F*(2, 65) = 9.38, *p* < 0.001, η² = 0.22), but not for the left IFG (*F*(2, 65) = 1.99, *p* = 0.15), suggesting that the effect observed for the bilateral IFG was driven by the right IFG. Similarly, a significant effect of group was observed for the right DLPFC (*F*(2, 65) = 3.89, *p* = 0.025, η² = 0.11), but not for the left DLPFC (*F*(2, 65) = 2.54, *p* = 0.094), again suggesting that the effect observed for the bilateral DLPFC was driven by the right DLPFC. At the delayed time point, a significant effect of group was observed for the left IFG (*F*(2, 65) = 3.59, *p* = 0.033, η² = 0.10), and for the right IFG (*F*(2, 65) = 5.45, *p* = 0.006, η² = 0.14).

Exploratory comparison of the two time points within groups showed that rCBF in the IFG decreased from the acute to the delayed time point in the PK (*p* = 0.007), and in the LK (*p* = 0.002) group, but not in the PP (*p* = 0.28) group. In the DLPFC, a decrease of rCBF from the acute to the delayed time point was observed in the PK group (*p* = 0.017), while an increase was observed in the PP group (*p* = 0.022). In the LK group no difference between time points was observed (*p* = 0.21).

Exploratory correlation analyses conducted in the PK group between rCBF and subjective measures as well as plasma concentration showed the following results: At both time points significant correlations were only found between rCBF and subjective mood ratings. At the acute time point, a positive correlation was found between rCBF in the left DLPFC and positive affect after the ketamine infusion (*r* = 0.54, *p* = 0.008). Furthermore, a positive correlation was found between rCBF in the right DLPFC and negative affect before the ketamine infusion (*r* = 0.49, *p* = 0.018). At the delayed time point, a negative correlation was found between rCBF in the left DLPFC and negative affect prior to the scanning session (*r* = −0.47, *p* = 0.036). Interestingly, there was also a positive correlation between rCBF in the right DLPFC at the acute timepoint with negative affect both prior (*r* = 0.55, *p* = 0.006) and after (*r* = 0.49, *p* = 0.016) the scanning session at the delayed timepoint. Changes in perfusion were neither associated with dissociative and psychotomimetic effects nor with plasma concentration of ketamine.

## Discussion

To our knowledge, this is the first study to investigate not only acute and delayed effects of a single dose of ketamine on CBF, but also consequences of modulated glutamate release via lamotrigine pretreatment. Our findings demonstrate region- and time- specific effects of ketamine administration per se as well as of lamotrigine pretreatment. Compared to placebo, acute ketamine administration was associated with a higher perfusion in IFG and DLPFC, but not in ACC, amygdala, hippocampus, and thalamus. Inhibition of glutamate release by pretreatment with lamotrigine abolished ketamine’s effect on perfusion. Perfusion increases both in IFG and DLPFC occurred in the right, but not in the left hemisphere. At the delayed time point, there was no longer an effect of ketamine administration per se on IFG perfusion, while pretreatment with lamotrigine was associated with lower perfusion. Ketamine-induced CBF changes in the investigated regions were associated with mood, but not with dissociative and psychotomimetic phenomena nor with plasma concentration of ketamine.

Our findings regarding increased perfusion during ketamine administration are partly consistent with results of prior studies. However, while previous reports included widespread increases in CBF in prefrontal and cingulate cortices as well as in subcortical regions such as thalamus [8, 11–15], our findings point to more region- specific effects with increases in DLPFC and IFG, but not in ACC and thalamus. Also, we could not replicate the finding of CBF decrease in hippocampus or in adjoining amygdala. Perfusion increases measured in DLPFC and IFG are consistent with results of several PET studies reporting increased glucose metabolism and perfusion in frontal and insula areas in healthy volunteers [17–19, 44, 45]. In preclinical studies, glutamatergic neurotransmission plays a key role in the regulation of CBF by activating NMDA receptors on neurons and metabotropic glutamate receptors on astrocytes, with the subsequent rise in intracellular Ca2+ leading to the release of intracellular-vasodilating messengers [46]. Our findings thereby underscore the idea that regionally selective patterns of CBF changes reflect proximate effects of modulated glutamate release on neuronal activity.

Results indicated large effects of acute ketamine administration on IFG perfusion. IFG and adjacent anterior insula (AI) are part of the salience network and have been proposed as a hub that ensures dynamic switching between external and internal control modalities [47–49]. Activity particularly in right IFG/ AI has also been associated with attention to interoceptive states and emotional processing [50, 51]. It has been proposed that based on interoceptive, emotional, and sensory inputs to these regions, an integrated representation of an emotional experience, i.e. awareness of the immediate moment, is formed [52, 53]. Increased CBF during ketamine administration might therefore reflect increased interoceptive and/ or emotional awareness. A recent study reported increased activity in inferior frontal cortex, comprising AI and IFG (pars triangularis and pars opercularis), during perceptual conflicts caused by ambiguous sensory information [54]. However, it seems unlikely that the here observed CBF changes merely reflect perceptual distortions or other psychotomimetic phenomena during ketamine, since none of the measures assessing their subjective experience was associated with CBF.

Ketamine administration also led to CBF increases in the DLPFC, a part of the cognitive control network and crucially involved in executive functions and emotion regulation [55, 56]. Prior BOLD imaging studies in healthy participants reported significant effects of ketamine on resting state activity in DLPFC [26, 57] and it has been proposed that the antidepressant effects of ketamine administration are mediated by targeting regions that subserve cognitive processing relevant to executive function and cognitive- emotional interaction [58, 59]. Again, CBF increases occurred only in the right DLPFC, which might be considered in light of the valence hypothesis, which states that right and left prefrontal cortex are dominant in the processing of negative and positive emotions, respectively [60]. Accordingly, higher perfusion in left DLPFC was correlated with more positive affect, while higher perfusion in right DLPFC was associated with more negative affect. Effects of ketamine on frontal brain functions have also been linked to its psychotomimetic effects [26, 61]. However, as for the IFG, there was no association between subjective experience and CBF.

Administration of ketamine results in a surge of glutamate [62, 63] and the few existing previous studies on the prolonged impact of ketamine administration on CBF reported diminished effects after 4 h in healthy participants [8, 11]. Consistently, our investigation of both acute and delayed effects showed that perfusion in IFG as well as in DLPFC decreased 24 h later compared to during ketamine administration. Interestingly, at the delayed time point, pretreatment with lamotrigine was associated with even lower perfusion in IFG. In the DLPFC, perfusion did not differ between groups at the delayed time point. Notably, previous imaging studies in depressive patients demonstrated delayed effects of ketamine in AI, that were associated with symptom improvement [64, 65]. In a recent study [22], reported CBF decreases in right AI after serial ketamine infusions in depressive patients. In light of our findings, one might therefore assume a temporal gradient of functional neuroplasticity after ketamine administration. Our results showed that lamotrigine pretreatment selectively abolished CBF increases in IFG and DLPFC during ketamine administration and thereby contradict findings of a prior ASL study [15]. Here, no effect on resting brain perfusion was found, which was seen as an indicator that the attenuation of ketamine’s acute effects by lamotrigine was not due to changes in neurovascular responsivity but rather to reduced glutamate release. However, as preclinical studies demonstrated that glutamatergic neurotransmission plays a key role in the regulation of CBF [46], one might assume that region- specific changes found here mirror the effect of modulated glutamate release on neuronal activity in these areas. Correspondingly, previous studies demonstrated an attenuation of ketamine’s acute effects on region- specific BOLD responses and connectivity by lamotrigine [26–28].

Results of our previous study in depressive patients also demonstrated an increase in thalamus perfusion 24 h after ketamine administration, that was associated with greater improvement of depressive symptoms [20]. Based on these results we also investigated CBF in the thalamus but found no acute or delayed effects of ketamine in healthy participants. It has been hypothesized, that decreased thalamic perfusion might reflect a disease- specific dysfunction of thalamico- cortical circuits in depression [66], while an increase mirrors a restoration of “normal” perfusion and is associated with symptom improvement [20–22]. Our results indicate that the effects of ketamine on thalamic perfusion might be specific to MDD patients. Similarly, CBF in ACC and amygdalo-hippocampal complex was not affected by ketamine administration, even though several prior studies reported CBF increases and decreases, respectively, in these regions [8, 11, 15]. The amygdalo- hippocampal complex and its projection to the ACC form an important affective neurocircuitry for mood regulation and show aberrant activation and connectivity in depressive patients [67]. Therefore, one might assume that previously reported CBF changes after ketamine in these regions are either specific to depressive patients or due to different analysis approaches, i.e., voxel-based and region of interest (ROI) analyses.

There are several limitations to this study. Previous reports described strongest effects of ketamine soon after beginning of the infusion [26, 68], while here imaging occurred approximately after 30 min of continuing ketamine infusion. This, however, reflects the steady state of the brain well after the intense immediate action of ketamine and our data clearly demonstrate profound effects of ketamine on rCBF as well as on subjective measures. Our results revealed no association of perfusion with psychotomimetic effects, while region specific changes in CBF could be linked positive and negative mood, respectively. Thereby, this finding rather argues against changes in perfusion reflecting neurophysiological changes associated with psychotomimetic phenomena.

To conclude, we here provide first evidence of region- and time- specific effects of modulated glutamate release on perfusion. The increase of perfusion in IFG and DLPFC during acute ketamine administration was abolished by inhibition of glutamate release via pretreatment with lamotrigine, which furthermore led to decreased IFG perfusion 24 h later. These findings underscore the idea that regionally selective patterns of CBF changes reflect proximate effects of modulated glutamate release on neuronal activity. Furthermore, region- specific sustained effects indicate both a swift restoration of disturbed homeostasis in DLPFC as well changes occurring beyond the immediate effects on glutamate signaling in IFG.

### Supplementary information

Supplementary Material

Consort Flowchart

## Data Availability

All data generated or analysed during this study are included in this article. Further enquiries can be directed to the corresponding author.
