# Supplementary material for: Region- and time- specific effects of ketamine on cerebral blood flow: a randomized controlled trial
Source: Neuropsychopharmacology. 2023 May 25;48(12):1735–41. doi: 10.1038/s41386-023-01605-4 (PMC10579356; doi:10.1038/s41386-023-01605-4)
Supplement: Supplementary file 1 — Supplementary Material [file 41386_2023_1605_MOESM1_ESM.docx]

**Supplementary Methods**

Sample Size

Power analysis for a one-way ANOVA with 3 groups was conducted in G*Power (Faul et al., 2007) to determine a sufficient sample size using an alpha of 0.05, a power of 0.80, and a large effect size of f = 0.4. This effect size (averaged for acute and delayed effects of ketamine as both are primary endpoints) was based on previously reported effect sizes (d = 0.815 for acute effects and d = 0.776 for delayed ketamine effects, Abdallah et al. 2018), showing a significant increase in prefrontal global connectivity during infusion and at 24-h posttreatment as compared to placebo. The sample size needed with this effect size was N = 66 (22 subjects per group). Accounting for a drop-out rate of ∼15 %, the estimated total sample size was N = 75 (25 subjects per group). Screening for the trial was stopped once a sufficient number of subjects had been measured and controlled for dropouts due to excessive head movement in the scanner and/or headaches. Total study duration was from March 05, 2020 to December 10, 2020.

Randomization

Randomization, enrollment of participants, and assignment to interventions was conducted by the Charité Research Organisation (CRO), GmbH. After written informed consent, subjects meeting all in-/exclusion criteria were randomized at baseline to one of three experimental conditions (lamotrigine + ketamine, placebo + ketamine, placebo). The three treatments were allocated 1:1:1 randomization. Each subject received only one single dose of blinded combined study medication (lamotrigine + ketamine, placebo + ketamine or placebo + placebo) in the sequence according to randomization administered by the site staff. Randomization numbers were assigned in ascending, sequential order to eligible subjects. Additional numbers were used in case of replacements being needed. The investigator documented the randomization number in the eCRF. The randomization list was kept in safe and confidential custody. Only personnel not involved in the study had access to the list.

Blinding

For the conduct of this study, the study drugs as well as matching placebos (lamotrigine + ketamine, placebo + ketamine or placebo + placebo) were administered in a double-blind fashion at the site. Doses were prepared by an unblinded member of the study team. This unblinded member was not involved in any study assessments. The identity of the treatments was concealed by using study drugs that were all identical in packaging, labeling, schedule of administration, appearance, and odor. Only the unblinded site team had access to the unblinded randomization list. Subjects remained blinded to study treatment throughout the study. Anyone who was involved in subject -related assessments was blinded with regards to the treatment assigned.

Harms

Safety assessments included vital signs, physical examinations, ECGs, standard clinical laboratory evaluations (hematology, blood chemistry, and coagulation), adverse events and serious adverse event monitoring.

Adverse events

No severe adverse events occurred. A detailed overview of all minor adverse events can be found in Supplementary Table 1 (see below). At screening appointments, a total of n = 3 minor pretreatment adverse events were noted: Respectively one participant was suffering from a skin rash, herpes labialis and a hordeolum.

Vital signs

Vital signs included pulse rate, systolic and diastolic blood pressure, and body temperature. Body temperature was measured in the ear. Vital signs could be recorded at any time, if medically imperative for clarification of clinical signs and symptoms. For n = 35 participants abnormal vital signs were reported; body temperature, blood pressure or pulse rate were out of range. None of the abnormal vital signs were clinically significant, except for one participant who, however, reported to feel well. During the course of the study, none of the participants was excluded due to abnormal vital signs. A detailed description of all abnormal vital signs can be presented on request.

Headache severity

A total of n = 16 participants reported headaches either prior or after fMRI assessment, mean NRS score was = 1.4, all NRS scores were < 5, thus none of the participants was excluded due to reported headache.

Electrocardiogram

For n = 3 participants abnormal ECG values were detected. In accordance with the study protocol, measurements were repeated. During the repeated measurements, no abnormal values were detected.

Physical examination

Information for all physical examinations was included in the source documentation at the study site, only medically relevant findings were recorded in the eCRF. For examinations at the Screening visit, medically relevant findings were also captured as medical history in the eCRF.

Deaths and other serious adverse events

No AEs leading to discontinuation or other significant AEs occurred during the study.

Adverse Events leading to discontinuation and other significant AEs

One subject discontinued study participation due to a panic reaction in the scanner.

Clinical laboratory evaluation

The following parameters were assessed: sodium, postassium, calcium, magnesium, total protein, albumin, glucose, creatinine, urea, bilirubin, AST, ALT, GGT, LDH, AP, CRP; hematology: leukocytes, granulocytes, neutrophils, eosinophils, basophiles, lymphocytes, monocytes, erythrocytes, thrombocytes, haematocrit, haemoglobin; oagulation: aPTT, INR.

Supplementary information on the pre-study used for the ROI definition

*Overall concept* Next to testing the logistical implementation of the fMRI setup for subsequent studies, the main objective of this pre-study was to evaluate whether a set of emotional face and scene images reliably evoked BOLD signal changes in regions of interest. The resulting activation maps were then used to create an unbiased set of regions of interest for use in subsequent studies. For this purpose, 15 healthy male subjects, age 18 to 45 years, participated in an MRI session that included two fMRI tasks: a Face Processing Task and a Scenes Processing task. A description of these tasks and their use in subsequent clinical research can be found in Paret et al. (2021). The fMRI tasks were previously also used in Paret et al. (2014).

*Tasks* In the first task, participants viewed faces with emotional expressions from the Warsaw Set of Emotional Facial Expression Pictures (http://www.emotional-face.org/). A block design of 12 blocks with 6 faces each (aversive condition [AC]; negative emotional expressions were randomly mixed within blocks) and 12 blocks with scrambled faces (neutral condition [NC]) was used. In sum, 72 negative faces of 24 actors (12 female, 12 male) were shown for 3 seconds each. To ensure attention, participants were asked to press a button to indicate for every picture whether the person was male or female (AC) or whether the color of the bounding box around the scrambled faces was blue or green (NC). The second task was adapted from Paret et al. (2014). Pictures with negative affective valence and high arousal (AC) from the OASIS picture set (Kurdi et al., 2017) were presented in a block design to induce negative affect. During each of the 14 blocks, lasting 18 seconds, 3 picture stimuli were presented for 6 seconds each, resulting in a set of 42 negative pictures in total. Scrambled pictures were used in a non-affective control condition (NC) with the same number of trials and presentation time. To ensure attention, participants were asked to press a button to indicate for every picture whether it showed a person or not (AC) or whether the color of the bounding box around scrambled pictures was blue or green (NC).

*Preprocessing* Both tasks were analyzed and preprocessed using identical pipelines. All imaging preprocessing and first-level analyses were carried out using FEAT Version 6.00, part of FSL (www.fmrib.ox.ac.uk/fsl). The following preprocessing steps were performed: volume realignment to correct for participant head motion, B0 unwarping using fieldmap data to correct for echo-planar imaging distortions, grand-mean scaling, and spatial smoothing with a 5-mm full width at half maximum kernel. Next, FSL’s melodic was applied to extract independent data components, and ICA-AROMA (Pruim et al., 2015) was applied to identify and remove secondary effects of head motion. Finally, a temporal high-pass filter with 0.01 Hz cutoff was applied to remove scanner drifts. We obtained the transformation of the fMRI data to the participant’s high-resolution T1 anatomical space using FSL’s Boundary-Based Registration tool. A transformation from the participant’s T1 space to MNI152 standard space was obtained using linear alignment via FSL FLIRT with 12 degrees of freedom and subsequently refined using nonlinear steps as implemented in FSL FNIRT.

*Statistical Analysis* After preprocessing, we conducted individual-level first-level statistical analyses for both the faces and the scenes tasks separately. For each task, we included two regressors, respectively, modeling the onset times of the faces/scenes (AC) and scrambled stimuli (NC), convolved with a double-gamma hemodynamic response function. The onset regressors consisted of 18-second blocks. The contrast of interest compared BOLD activity between the scenes/faces and the scrambled control stimuli. For each task first-level statistical maps were subsequently included in a second-level group analysis that modelled activity across the 16 participants using a one-sample T-test. Resulting group-level statistical images were thresholded at z>3.1 and a (corrected) cluster significance threshold of P<0.05. The figures below show the group-level activation maps for the Faces and Scenes tasks (see Supplementary Figure 1 & 2).

*ROI construction* Group-level activation maps for FACES > NEUTRAL and SCENES > NEUTRAL were used to construct regions of interest for subsequent independent studies that would use the Faces and/or Scenes tasks in their protocol. To this end we summarized both thresholded activation maps into one binarized conjunction map. To increase anatomical specificity the binarized conjunction map was multiplied with binarized regions from the Harvard-Oxford Atlas (HOA) included with FSL. The atlas regions of interest were: middle frontal gyrus (for ROI Dorsolateral Prefrontal Cortex), paracingulate gyrus (for ROI Anterior Cingulate Cortex), and inferior frontal gyrus (for ROI Inferior Frontal Gyrus). Prior to multiplication, the HOA probability masks were thresholded to include only voxels with a probability exceeding 10%. After creation of the final binarized ROI masks, the masks for DLPFC and IFG were split into a left and right region using a midline split (see Supplementary Figure 3).

Control analyses including sex as covariate

Findings on the effect of sex on cerebral blood flow are inconsistent, with prior studies reporting higher CBF values in women (Alisch et al., 2021), no differences between men and women (Smith et al., 2019) as well as age- related CBF decreases in women (Liu et al., 2016). To account for these inconsistencies, we re- analyzed our data with sex as a covariate, which did not affect our findings significantly (see Supplementary Table 4 for comparison group effects with and without covariate).


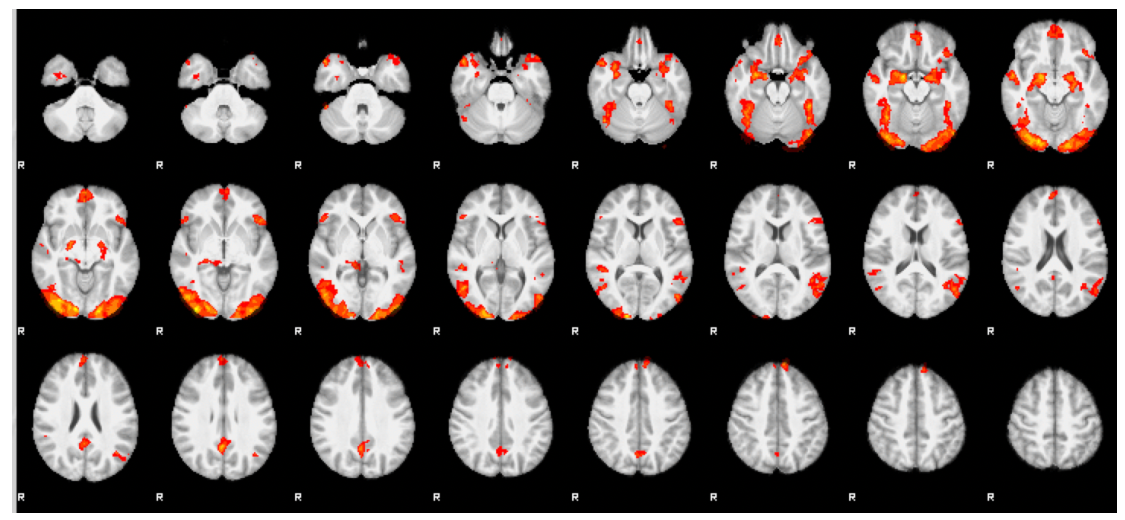


Supplementary Figure 1: Group-level activation map for FACES > NEUTRAL from a one-sample t-test.


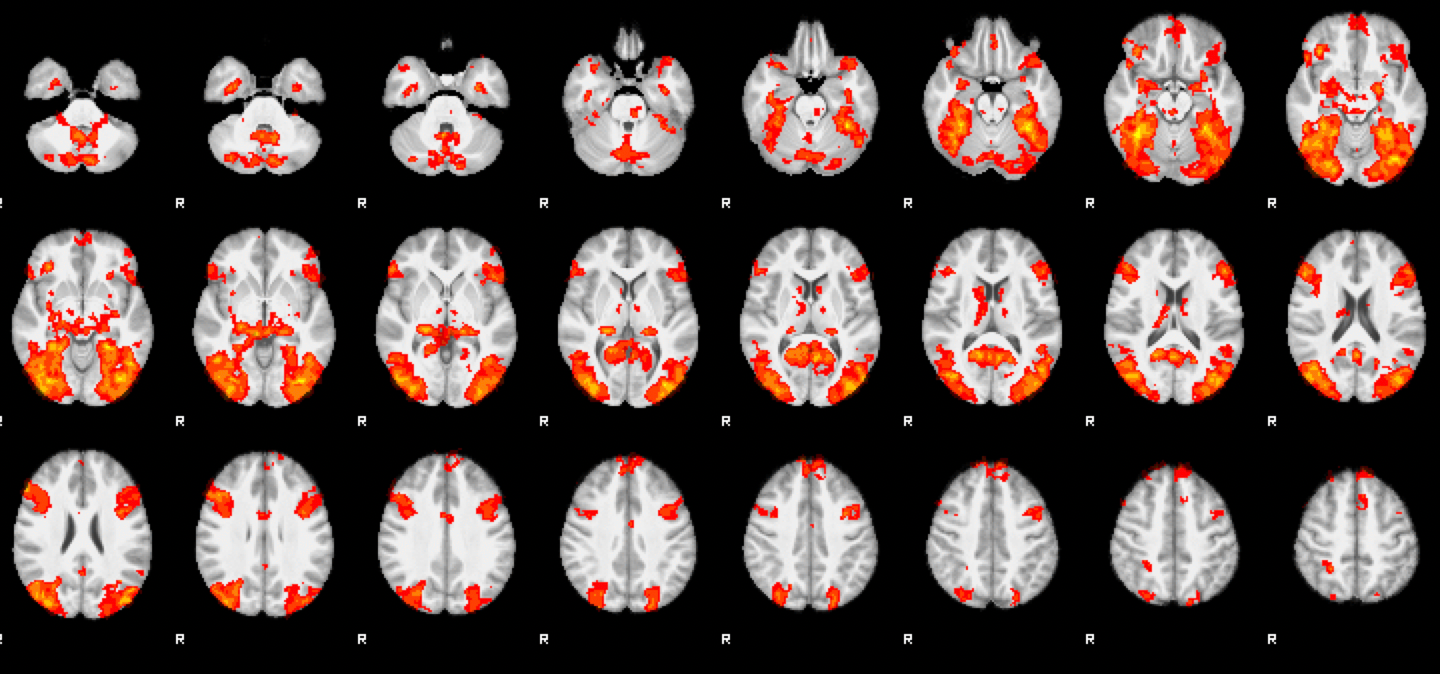


Supplementary Figure 2: Group-level activation map for SCENES > NEUTRAL from a one-sample t-test.


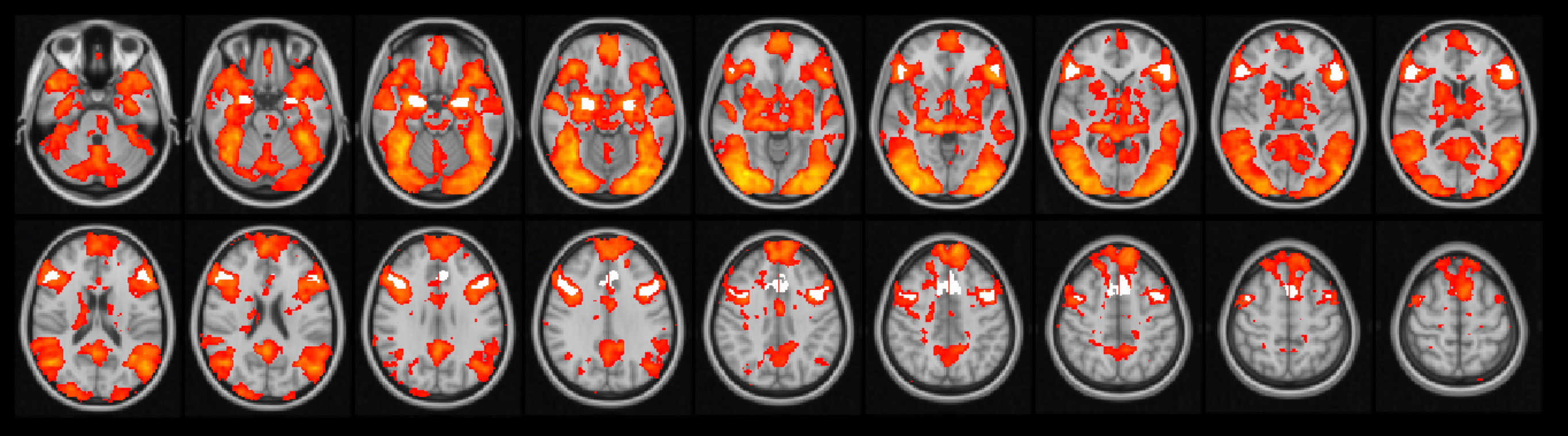


Supplementary Figure 3: Final ROIs (white) overlayed on top of the conjunction activation map.

**Supplementary Table 1**

*Adverse events (AE).*

| Adverse Events | Number of AE visit 1 | | | Number of AE visit 2 | | |
| --- | --- | --- | --- | --- | --- | --- |
|  | PP | PK | LK | PP | PK | LK |
| *Headache* | 10 | 8 | 9 | 2 | 2 | 3 |
| *Dizziness* | 0 | 8 | 5 | 0 | 0 | 0 |
| *Urinary tract infection* | 1 | 0 | 0 | 0 | 0 | 0 |
| *Difficulty concentrating* | 0 | 2 | 1 | 0 | 0 | 0 |
| *Nausea* | 2 | 3 | 1 | 0 | 0 | 0 |
| *Feeling of numbness* | 0 | 3 | 3 | 0 | 0 | 0 |
| *Vomiting* | 1 | 2 | 1 | 0 | 0 | 0 |
| *Blurred vision* | 0 | 0 | 1 | 0 | 0 | 0 |
| *Changed visual perception* | 1 | 0 | 0 | 0 | 0 | 0 |
| *Fatigue* | 0 | 1 | 2 | 0 | 0 | 0 |
| *Sore throat* | 0 | 0 | 0 | 1 | 0 | 0 |
| *Panic reaction* | 0 | 0 | 1 | 0 | 0 | 0 |
| *Back pain* | 1 | 0 | 0 | 0 | 0 | 0 |
| *Hematoma* | 0 | 1 | 0 | 0 | 0 | 0 |
| *Drowsiness* | 1 | 0 | 0 | 0 | 0 | 0 |

*Notes.* PP = Placebo-Placebo, PK = Placebo-Ketamine, LK = Lamotrigine-Ketamine.

**Supplementary Table 2**

*Detailed description of the main ROIs.*

|  | Left | | | | |  | Right | | | | |
| --- | --- | --- | --- | --- | --- | --- | --- | --- | --- | --- | --- |
|  | x | y | z | volume (mm3) | volume  (n voxels) |  | x | y | z | volume (mm3) | volume  (n voxels) |
| ACC | -5 | 20 | 41 | 4120 | 515 |  | 6 | 17 | 44 | 2376 | 297 |
| DLPFC | -43 | 13 | 36 | 4488 | 561 |  | 44 | 15 | 34 | 3256 | 407 |
| IFG | -49 | 29 | 8 | 4344 | 543 |  | 51 | 29 | 8 | 3952 | 494 |

*Notes.* ACC, anterior cingulate cortex; DLPFC, dorsolateral prefrontal cortex; IFG / AI, inferior frontal gyrus / anterior insula; x, y, z depict the center of gravity coordinates in MNI space.

**Supplementary Table 3**

*Baseline demographics for each group.*

|  | **PK** | **LK** | **PP** | **Group statistic** |
| --- | --- | --- | --- | --- |
|  | *M (SD)* | *M (SD)* | *M (SD)* |  |
| Age | 28.65 (6.51) | 26.7 (5.06) | 30.45 (6.82) | *F*(2, 65) = 2.09, *p* = .13 |
| Sex (F:M) | 5:18 | 11:12 | 8:14 | *X²*(2, 68) = 3.44, *p* = .18 |
| Ketamine plasma concentration | 106.78 (28.08) | 92.28 (32.45) | - | *T*(44) = 1.62, *p* = .11 |

*Notes.* PK, placebo-ketamine; LK, lamotrigine-ketamine; PP, placebo-placebo

**Supplementary Table 4**

*Control analysis including sex as covariate.*

| ROI | ANOVA | ANCOVA |
| --- | --- | --- |
| IFG  DLPFC  ACC | 0.002**  0.012*  0.48 | 0.008**  0.005**  0.52 |

**References**

Alisch, J. S. R., Khattar, N., Kim, R. W., Cortina, L. E., Rejimon, A. C., Qian, W., Ferrucci, L., Resnick, S. M., Spencer, R. G., & Bouhrara, M. (2021). Sex and age-related differences in cerebral blood flow investigated using pseudo-continuous arterial spin labeling magnetic resonance imaging. *Aging*, *13*(4), 4911–4925. https://doi.org/10.18632/aging.202673

Faul, F., Erdfelder, E., Lang, A.-G., & Buchner, A. (2007). G*Power 3: A flexible statistical power analysis program for the social, behavioral, and biomedical sciences. *Behavior Research Methods*, *39*(2), 175–191. https://doi.org/10.3758/BF03193146

Kurdi, B., Lozano, S., & Banaji, M. R. (2017). Introducing the Open Affective Standardized Image Set (OASIS). *Behavior Research Methods*, *49*(2), 457–470. https://doi.org/10.3758/s13428-016-0715-3

Liu, W., Lou, X., & Ma, L. (2016). Use of 3D pseudo-continuous arterial spin labeling to characterize sex and age differences in cerebral blood flow. *Neuroradiology*, *58*(9), 943–948. https://doi.org/10.1007/s00234-016-1713-y

Paret, C., Kluetsch, R., Ruf, M., Demirakca, T., Kalisch, R., Schmahl, C., & Ende, G. (2014). Transient and sustained BOLD signal time courses affect the detection of emotion-related brain activation in fMRI. *NeuroImage*, *103*, 522–532. https://doi.org/10.1016/j.neuroimage.2014.08.054

Paret, C., Niedtfeld, I., Lotter, T., Wunder, A., Grimm, S., Mennes, M., Okell, T., Beckmann, C., & Schmahl, C. (2021). Single-Dose Effects of Citalopram on Neural Responses to Affective Stimuli in Borderline Personality Disorder: A Randomized Clinical Trial. *Biological Psychiatry: Cognitive Neuroscience and Neuroimaging*, *6*(8), 837–845. https://doi.org/10.1016/j.bpsc.2021.02.002

Pruim, R. H. R., Mennes, M., van Rooij, D., Llera, A., Buitelaar, J. K., & Beckmann, C. F. (2015). ICA-AROMA: A robust ICA-based strategy for removing motion artifacts from fMRI data. *NeuroImage*, *112*, 267–277. https://doi.org/10.1016/j.neuroimage.2015.02.064

Smith, L. A., Melbourne, A., Owen, D., Cardoso, M. J., Sudre, C. H., Tillin, T., Sokolska, M., Atkinson, D., Chaturvedi, N., Ourselin, S., Hughes, A. D., Barkhof, F., & Jäger, H. R. (2019). Cortical cerebral blood flow in ageing: Effects of haematocrit, sex, ethnicity and diabetes. *European Radiology*, *29*(10), 5549–5558. https://doi.org/10.1007/s00330-019-06096-w
