# Supplementary figures and images for: Region- and time- specific effects of ketamine on cerebral blood flow: a randomized controlled trial
Source: Neuropsychopharmacology. 2023 May 25;48(12):1735–41. doi: 10.1038/s41386-023-01605-4 (PMC10579356; doi:10.1038/s41386-023-01605-4)

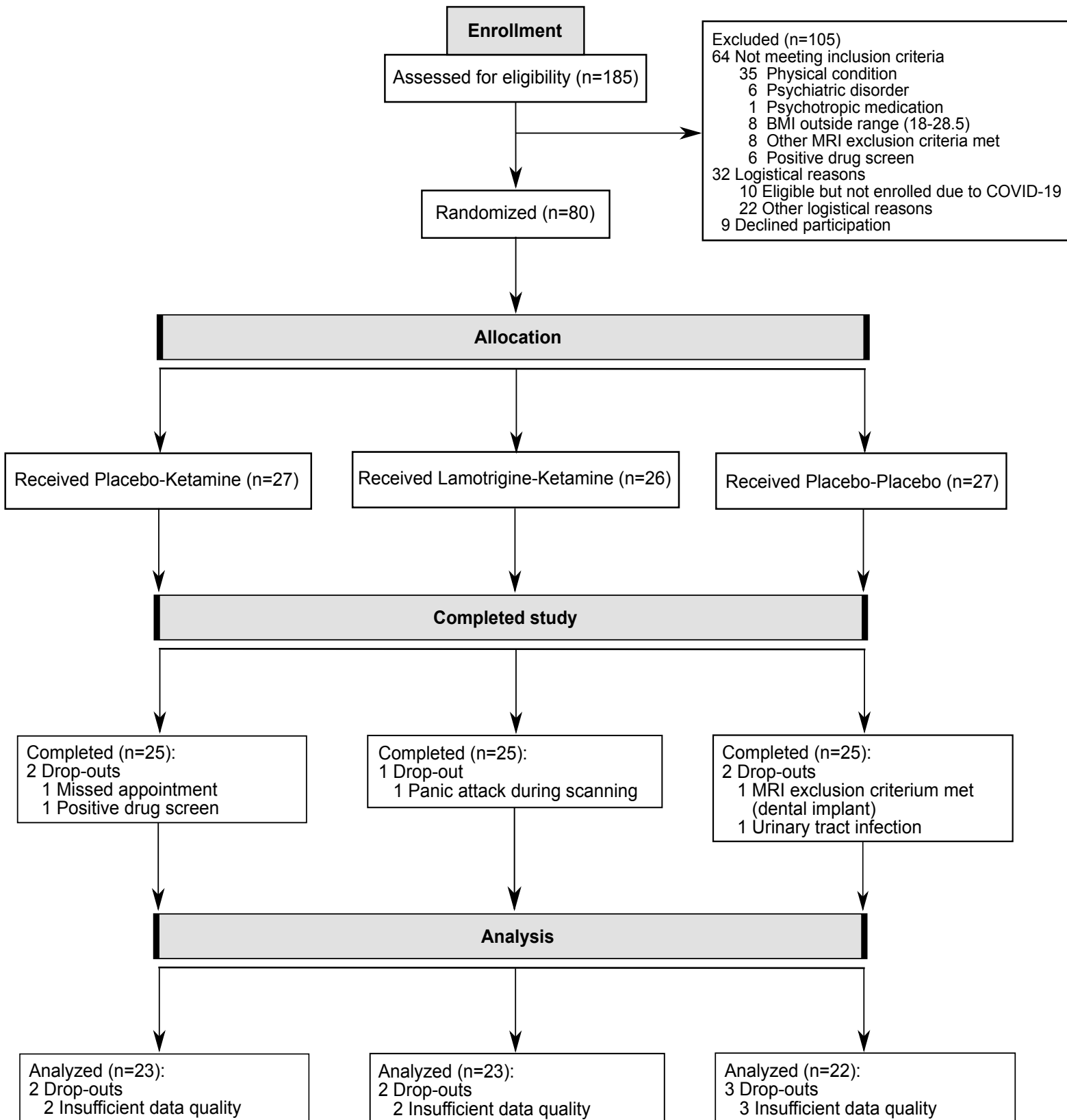

Supplement: Supplementary file 2 — Consort Flowchart [file 41386_2023_1605_MOESM2_ESM.pdf]
